# Advances in synthetic approach to and antifungal activity of triazoles

**DOI:** 10.3762/bjoc.7.79

**Published:** 2011-05-25

**Authors:** Kumari Shalini, Nitin Kumar, Sushma Drabu, Pramod Kumar Sharma

**Affiliations:** 1 Department of Pharmaceutical Technology, Meerut Institute of Engineering & Technology, Meerut, U. P., India, Pin-250005; 2 Director, M.S.I.P., Janakpuri, New Delhi, India

**Keywords:** antifungal, aspergillosis, candidiasis, cryptococcal meningitis, triazole

## Abstract

Several five membered ring systems, e.g., triazole, oxadiazole dithiazole and thiadiazole with three heteroatoms at symmetrical or asymmetrical positions have been studied because of their interesting pharmacological properties. In this article our emphasis is on synthetic development and pharmacological activity of the triazole moiety which exhibit a broad spectrum of pharmacological activity such as antifungal, antibacterial, anti-inflammatory and anticancer etc. Triazoles have increased our ability to treat many fungal infections, for example, candidiasis, cryptococcal meningitis, aspergillosis etc. However, mortality due to these infections even with antifungal therapy is still unacceptably high. Therefore, the development of new antifungal agents targeting specific fungal structures or functions is being actively pursued. Rapid developments in molecular mycology have led to a concentrated search for more target antifungals. Although we are entering a new era of antifungal therapy in which we will continue to be challenged by systemic fungal diseases, the options for treatment will have greatly expanded.

## Review

### Introduction

The alarming rates of the growing emergence of antimicrobial resistance are major concerns to the public health and scientific communities worldwide, especially in the field of multidrug- resistant bacteria and fungi [1–2]. These trends have emphasized the urgent need for new, more effective, less toxic and safe antimicrobial agents and the development of structurally new classes of antimicrobials with novel mechanisms of action as well as structural modifications to improve both their binding affinity and their spectrum of activity. One such strategy that has been pursued in recent years with increasing significance employs a combination of two different active fragments in one molecule [3]. With this strategy, various drug moieties have been designed to bind independently to different biological targets to produce beneficial effects [4]. The chemistry of N-bridged heterocyclic compounds, such as triazole, has received considerable attention in recent years due to their biological activities. Triazole is one of a pair of isomeric chemical compounds with the molecular formula C_2_H_3_N_3_. It is a basic aromatic heterocyclic ring [5]. Triazole derivatives are known to exhibit various pharmacological properties such as antimicrobial [6–10], antitubercular [11], anticancer [12–13], anticonvulsant [14], anti-inflammatory, analgesic [15] and antiviral [16]. Triazoles have also been incorporated in a wide variety of therapeutically interesting drugs including H_1_/H_2_ histamine receptor blockers, CNS stimulants, anti-anxiety agents and sedatives [17]. The most important use, however, is as antimycotics in drugs such as fluconazole, itraconazole and voriconazole [18–19].

The triazole moiety is stable to metabolic degradation and capable of hydrogen bonding, which could be favorable in binding biomolecular targets as well as in increasing solubility [20]. Moreover, triazoles can function as attractive linker units which could connect two pharmacophores to give an innovative bifunctional drug, and thus have become increasingly useful and important in constructing bioactive and functional molecules [21–23]. Notably, the bioisosteric replacement between triazole moiety and its bioisoster triazole has received special attention in medicinal chemistry, which represented an efficient concept for the discovery and development of novel triazole drugs, significantly extending the chemical space of triazole scaffolds possessing potent activities or enhancing biological activities [24]. Additionally, many investigations have shown that the addition of alkyl chains and/or various aromatic substituents, especially containing halogen atoms, has an important effect on the antimicrobial activities. The antifungal azoles are a class of synthetic compounds that possess one or more azole rings. Whilst both imidazole and triazole are five membered ring heterocycles, imidazole contains two ring nitrogen atoms, whereas triazoles have three. However, compared with imidazoles (clotrimazole, ketoconazole, miconazole), triazoles are less susceptible to metabolic degradation and have much greater target specificity, increased potency and an expanded spectrum of activity [25–26].

### Chemistry of triazoles

**Figure 1:**
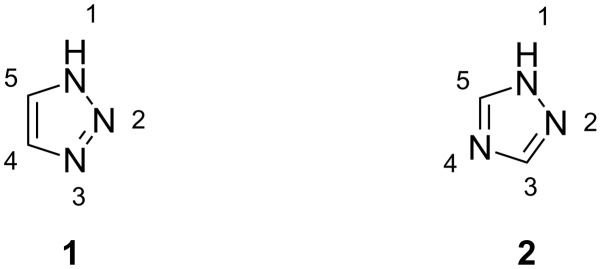
Isomeric forms of triazole.

Triazole refers to either one of a pair of isomeric chemical compounds with the molecular formula C_2_H_3_N_3_, and has a five membered ring containing two carbon and three nitrogen atoms. Triazoles have two isomeric forms, i.e., 1,2,3-triazole (**1**) and 1,2,4-triazole (**2**) (Figure 1).

Triazoles are basic aromatic heterocyclic compounds. 1,2,3-Triazoles are surprisingly stable compared to other organic compounds with three adjacent nitrogen atoms. However, flash vacuum pyrolysis at 500 °C leads to loss of molecular nitrogen (N_2_) to produce aziridine. Certain triazoles are relatively easy to cleave by ring–chain tautomerism.

#### Synthesis of triazoles

**Scheme 1:**
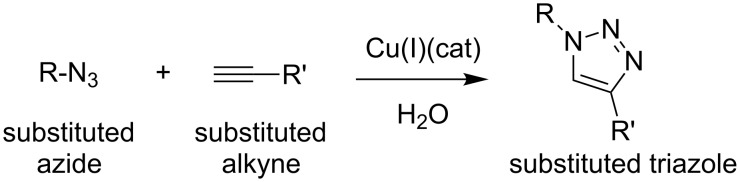
Copper catalyzed azide–alkyne cycloaddition.

**Scheme 2:**
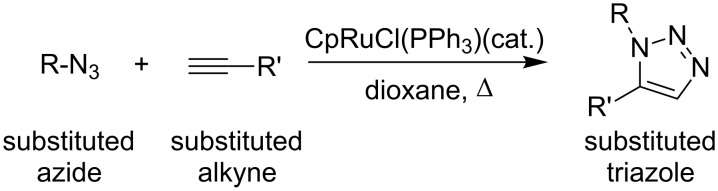
Ruthenium catalyzed azide–alkyne cycloaddition.

Substituted 1,2,3-triazoles can be produced by the azide–alkyne Huisgen cycloaddition in which an azide and an alkyne undergo a 1,3-dipolar cycloaddition reaction in the presence of a catalyst such as copper (Scheme 1) or ruthemium (Scheme 2) [27].

**Scheme 3:**
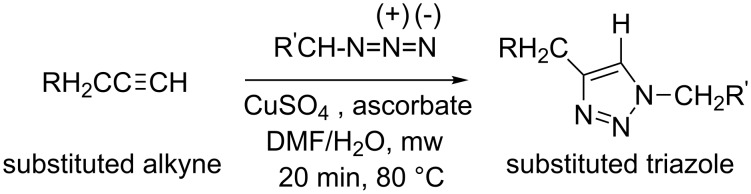
Copper-sulfate catalyzed azide–alkyne cycloaddition.

**Scheme 4:**
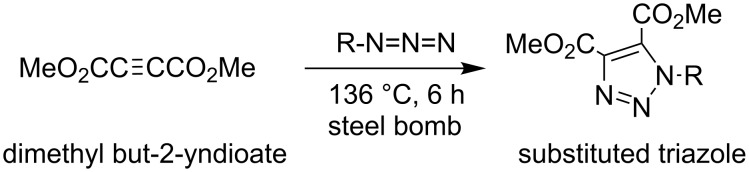
Azide–dimethylbut-2-yne-dioate cycloaddition.

Some new synthetic methods for 1,2,3-triazoles are given below (Scheme 3, Scheme 4) [28–29].

Structure activity relationships have revealed that bioisosteric replacement of a triazole ring leads to antifungal activity with a higher selectivity of the fungal targets. Triazole antifungal drugs are used in the treatment of both superficial and deep-seated candidiasis [30]. On the basis of various literature surveys, the triazole nucleus occupies the most important place in the treatment of fungal diseases. The triazole derivatives noted below were synthesized by various groups and show antifungal activity.

**Figure 2:**
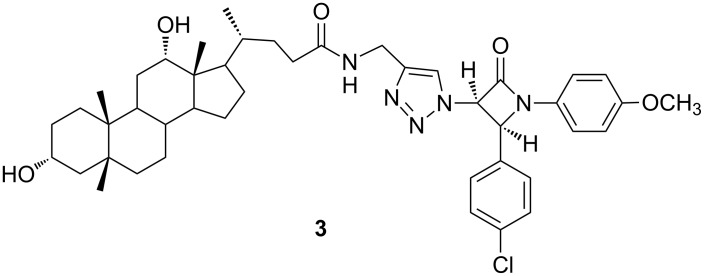
Triazole compound **3** with most potent antifugal activity against various strains [20].

Novel 1,2,3-triazole-linked with β-lactam–bile acid conjugates were prepared via 1,3-dipolar cycloaddition reactions of azido β-lactams and terminal alkynes of bile acids in the presence of a Cu(I) catalyst (click chemistry) by Vatmurge et al. The synthesized compounds were evaluated for their antifungal activity against different fungal strains such as *Candida albicans*, *Cryptococcus neoformans*, *Benjaminiella poitrasii, Yarrowia lipolytica* and *Fusarium oxysporum.* (4*R*)-*N*-((1-((2*S*,3*R*)-2-(4-chlorophenyl)-1-(4-methoxyphenyl)-4-oxoazetidin-3-yl)-1*H*-1,2,3-triazole-4-yl)methyl)-4-((3*R*,5*R*,10*R*,12*S*,13*R*)-hexadecahydro-3,12-dihydroxy-5,10,13-trimethyl-1*H*-cyclopenta[*a*]phenanthren-17-yl)pentanamide (**3,** Figure 2) had the most potent activity [20].

**Figure 3:**
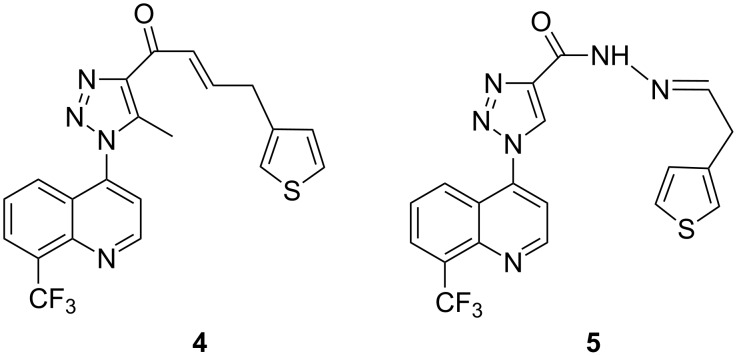
Triazole compounds **4** and **5** showing antifungal activity against *Candida albicans* [31].

Holla et al. synthesized triazole derivatives by the 1,3-dipolar cycloaddition reaction of 4-azido-8-trifluoromethylquinoline with ethyl acetoacetate and acetylacetone, respectively, and tested for antifungal activity against *Candida albicans*. Among the various synthesized compounds, 1-(1-(8-trifluoromethylquinolin-4-yl)-5-methyl-1*H*-1,2,3-triazole-4-yl)-4-(thiophen-3-yl)but-2-en-1-one (**4**) and 1-(8-trifluoromethylquinolin-4-yl)-*N*-(2-(thiophen-3-yl)ethylidene)-1*H*-1,2,3-triazole-4-carbohydrazide (**5**) (Figure 3) had the best antifungal activity [31].

**Figure 4:**
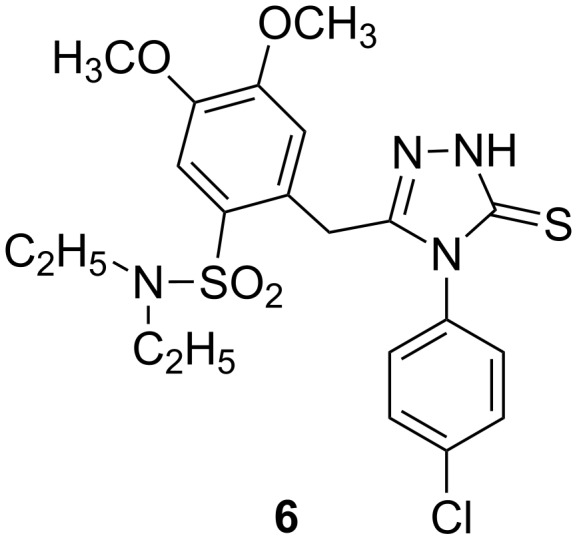
Triazole compound **6** with the highest activity against *Aspergillus flavus, Aspergillus versicolor, Aspergillus ochraceus, Aspergillus niger*, *Trichoderma viride* and *Penicillium funiculosum* [32].

A series of ten new 5-[2-(substituted sulfamoyl)-4,5-dimethoxy-benzyl]-4-aryl-1,2,4-triazole-3-thiones were synthesized by Ezabadi et al., and evaluated for in vitro antifungal activity against *Aspergillus flavus, Aspergillus versicolor, Aspergillus ochraceus, Aspergillus niger*, *Trichoderma viride* and *Penicillium funiculosum*. Of the compounds obtained, 5-[2-(*N*,*N*-diethylsulfamoyl)-4,5-dimethoxybenzyl]-4-(4-chlorophenyl)-1,2,4-triazole-3-thione (**6**, Figure 4) showed the highest activity [32].

**Figure 5:**
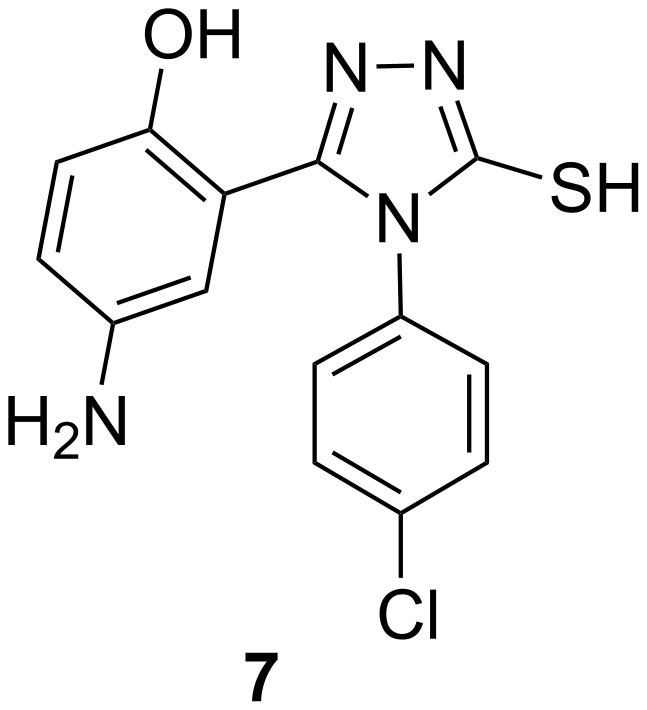
Triazole compound **7** exhibiting an MIC of 25 µg/mL against *Aspergillus niger* [33].

Various 4-amino-2-[4-(4-substituted phenyl)-5-sulfanyl-4*H*-1,2,4-triazol-3-yl] and 4-amino-2-{4-amino-5-[(4-substituted phenyl)amino]-4*H*-1,2,4-triazol-3-yl}phenol derivatives were synthesized by Hussain et al. and evaluated for their antifungal activity against *Aspergillus niger* by the cup plate method. 4-Amino-2-(4-(4-chlorophenyl)-5-mercapto-4*H*-1,2,4,-triazol-3-yl)phenol (**7**, Figure 5) with a chloro group at the para position of the phenyl ring exhibited a minimum inhibitory concentration (MIC) of 25 μg/mL against *Aspergillus niger* [33].

**Figure 6:**
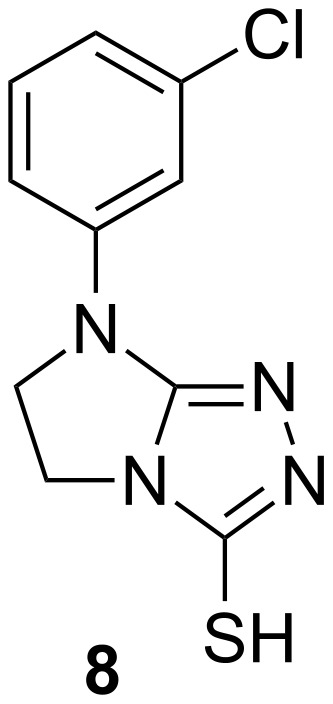
Triazole compound **8** showing the most significant activity against *Aspergillus niger* and *Fusarium oxysporum* [34].

Unsubstituted and 3-substituted-7-aryl-5*H*-6,7-dihydroimidazo[2,1-*c*][1,2,4]triazoles were designed by Sztanke et al. and obtained from biologically active 1-aryl-2-hydrazonoimidazolidines by cyclocondensation reactions with triethyl orthoformate, phenoxyacetic acid derivatives and carbon disulfide, respectively. Their antifungal activity was investigated against *Aspergillus niger* and *Fusarium oxysporum.* Among the series of synthesized compounds, 7-(3-chlorophenyl)-6,7-dihydro-5*H*-imidazo[2,1-*c*][1,2,4]triazole-3-thiol (**8**, Figure 6) showed the most significant activity [34].

### Antifungal chemotherapy

Fungal infections are caused by microscopic organisms that can invade the epithelial tissue. The fungal kingdom includes yeasts, moulds, rusts and mushrooms. Fungi are heterotrophic, which means they obtain nutrients from the environment, not from endogenous sources (like plants with photosynthesis). Fungal cells are complex organisms that share many biochemical targets with other eukaryotic cells. The fungal cell wall has unique organelles that produce toxicity. Systemic fungal infections are most important problems in phytopathology and infections caused by fungal species are most common in immune compromised patients [35]. Standard systemic antibiotic therapy alone is frequently unsatisfactory in certain cases, in addition more attention is being focused on addressing the problem of multi drug resistant bacteria. The emerging resistance of microorganisms to some synthetic antifungal agents makes it necessary to continue the search for new antimicrobial substances. Azoles are the largest class of antifungal agents in current clinical use [36]. During the last two decades, the incidence of invasive fungal infections (IFIs) increased dramatically worldwide [37–38]. IFIs are characterized by high morbidity and mortality and are difficult to diagnose, prevent and treat. In the USA, *Candida* spp. is the fourth most common nosocomial pathogen with the highest crude mortality rate (40%) [39]. In addition to *Aspergillus* spp., new and emerging fungal pathogens such as *Zygomycetes*, *Fusarium* spp. or *Scedosporium* spp. have become increasingly important pathogens. Their susceptibility to currently available antifungals may be limited and resulting mortality rate is ≥70% in patients with haematological malignancies [40]. For many years, the only available antifungal for IFIs was amphotericin B. With the introduction of triazoles at the beginning of 1990s, the pace of drug development accelerated. Amphotericin B (AMB) was incorporated in three lipid formulations, whilst the first-generation triazoles (fluconazole (**9**) and itraconazole (**10**)) changed the epidemiology of *Candida* infections and offered new treatment options. Many of these antifungals have proven to be less toxic and in some cases more effective than amphotericin B [41].

#### Mechanism of action

The antifungal triazoles (fluconazole (**9**), itraconazole (**10**), voriconazole (**11**), and currently investigated agents posaconazole (**12**) and ravuconazole (**13**)) are synthetic compounds that have one or more azole rings with three nitrogen atoms in a five membered ring. They act by inhibition of the cytochrome P450-dependent conversion of lanosterol to ergosterol [42]. Triazoles act as cytochrome P450 14α-demethylase inhibitors. This enzyme is involved in the conversion of lanosterol to ergosterol which is helpful in the cell wall synthesis. In this mechanism the basic nitrogen of the azole ring is tightly bound to the heme iron of the fungal cytochrome P450 preventing substrate and oxygen binding. Inhibition of the 14α-demethylase results in accumulation of sterols and causes permeability change and malfunction of membrane proteins.

**Figure 7:**
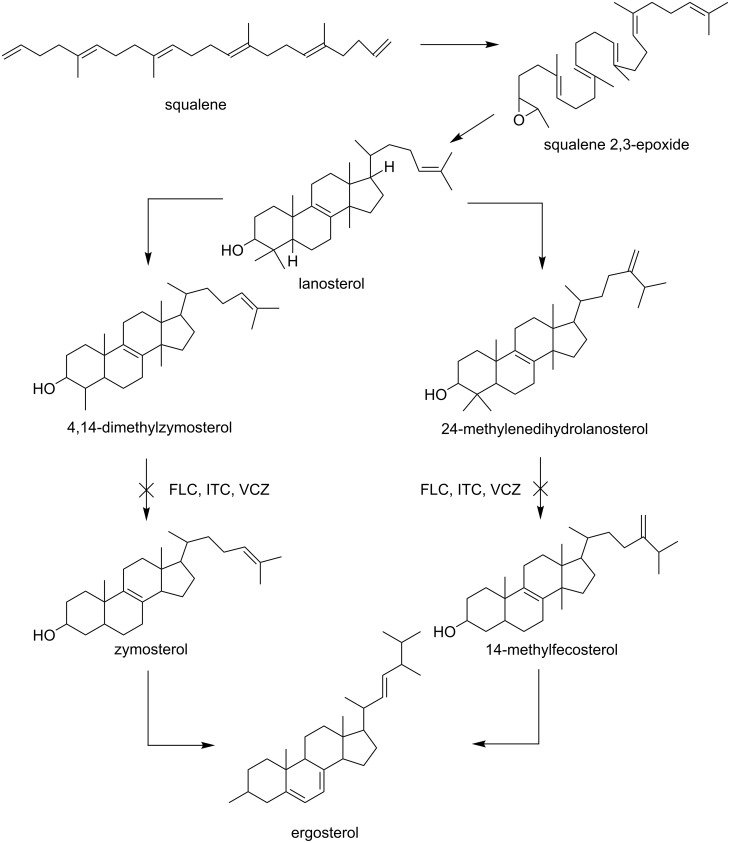
Ergosterol biosynthesis inhibitor pathway.

The ergosterol biosynthesis inhibitor pathway is shown in Figure 7 [43].

#### Classification of triazoles

**First generation of triazoles**

- Fluconazole (FLC, **9**)
- Itraconazole (ITC, **10**)

**Second generation of triazoles**

- Voriconazole (VCZ, **11**)
- Posaconazole (PCZ, **12**)
- Ravuconazole (RCZ, **13**)

#### First generation of triazoles

**Fluconazole**

**Figure 8:**
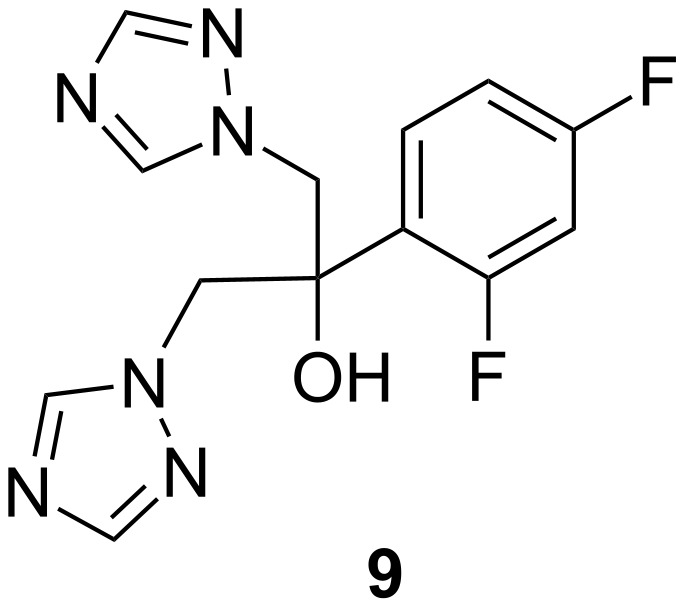
Fluconazole (**9**).

Fluconazole (**9**, Figure 8) is a fungistatic agent active against *Candida albicans, Candida tropicalis,* and *Candida glabrata*. Fluconazole is also used to treat meningitis, cryptococcal meningitis, systemic and mucosal candidiasis in both normal and immune compromised patients, coccidioidal meningitis, histoplasmosis and infections due to chemotherapy or radiation therapy prior to a bone marrow transplant [44–46].

**Itraconazole**

**Figure 9:**
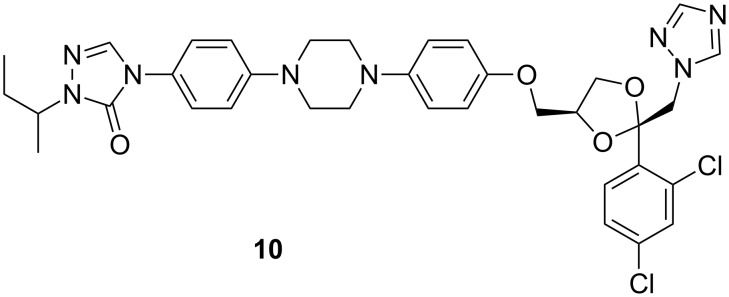
Itraconazole (**10**).

Itraconazole (**10**, Figure 9) has strain-dependent fungicidal activity against filamentous fungi with the exception of some strains of *Cryptococcus neoformans*. It is taken orally in capsule form to treat fungal infections that start in the lungs and spread throughout the body. Itraconazole can also be used to treat fungal infections of the nails. Oral solutions can be used to treat oral candidiasis [47–50].

Itraconazole produces some common side effects such as constipation, heartburn, bleeding gums, headache, and dizziness. More severe side effects can include: excessive tiredness, loss of appetite, nausea, vomiting, tingling or numbness in the extremities and difficulty in breathing or swallowing.

### Pharmacokinetics

#### First generation triazoles

The antifungal triazoles are generally well tolerated, but have significant potential for drug–drug interactions through their interference with cytochrome P450-dependent oxidative metabolism. Fluconazole (**9**) has nearly complete oral bioavailability, circulates in plasma as the free form, shows negligible hepatic metabolism, and is excreted unchanged through the kidneys. Itraconazole (**10**) is well absorbed by the gastrointestinal tract, high protein binding extensive hepatic metabolism, and is excreted in an inactive form via liver and kidneys. Fluconazole is 94% absorbed and its oral bioavailability is not affected by food or gastric pH. It is excreted unchanged in urine with t_1/2_ = 25–30 h. Itraconazole is largely metabolized in liver by cytochrome P450 3A4, an active metabolite is produced which is excreted in faeces; t_1/2_ = 30–64 h [42].

#### Second generation of triazoles

**Voriconazole**

**Figure 10:**
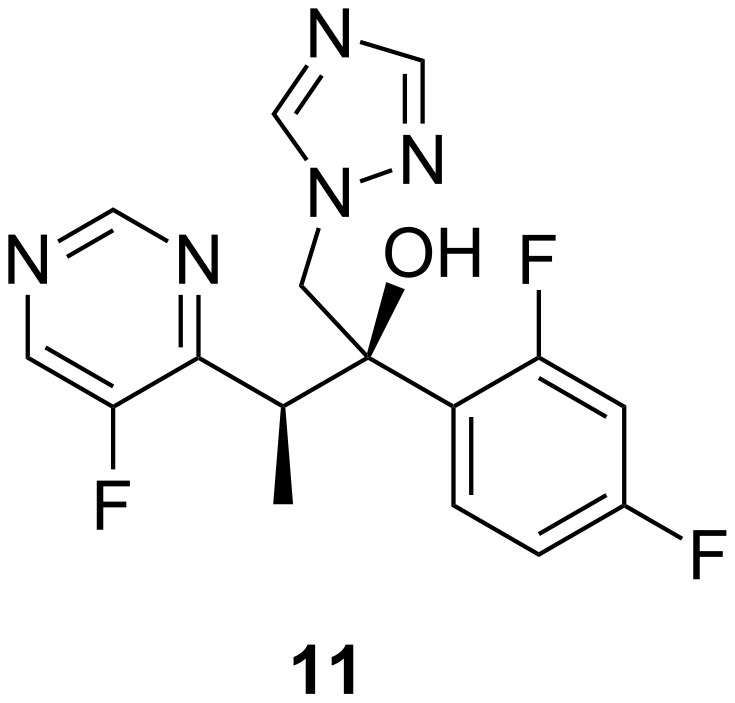
Voriconazole (**11**).

Voriconazole (**11**, Figure 10) was first marketed in 2002 and approved as a first-line treatment of oesophageal candidiasis, candidaemia, invasive aspergillosis, *Candida* infections of the skin, infections in the abdomen, kidney, bladder wall and wounds, and infections caused by *Scedosporium apiospermum* and *Fusarium* spp [51]. Voriconazole is available for both oral as well as intravenous (i.v.) administration and has excellent bioavailability (90%). It is metabolized in the liver and, in case of renal failure, its excretion is not affected. Its i.v. preparation is solubilized in sulfobutyl ether β-cyclodextrin sodium, which is secreted by the kidneys [52].

Voriconazole interacts with drugs that are substrates of cytochrome P450 3A4 (terfenadine, cisapride, etc.), by increasing their serum levels. It should be avoided in patients taking cyclosporine, rifampicin, carbamazepine, ritonavir and long-acting barbiturates.

Various studies have shown that voriconazole is fungistatic and has fungicidal activity against *Aspergillus* spp. [53]. Antifungal triazoles belong to the class of 14β-demethylase inhibitors, where antifungal activity is due to binding of this enzyme [54]. Voriconazole has good oral bioavailability but exhibits non-linear pharmacokinetics. Protein binding is low, cerebrospinal fluid levels exceed those of plasma levels several fold. Elimination occurs by oxidative hepatic metabolism and only small amounts of voriconazole are excreted unchanged into the urine.

**Posaconazole**

**Figure 11:**
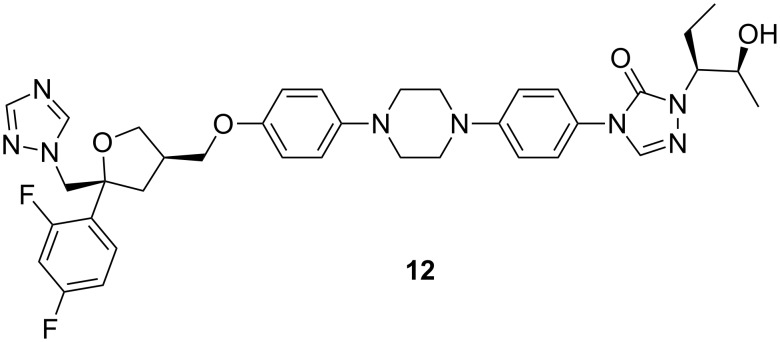
Posaconazole (**12**).

Posaconazole (**12**, Figure 11) is a hydroxylated analogue of itraconazole. It first became available in Europe in 2005 and it was approved by the Food and Drug Administration (FDA) in 2006 for prophylaxis against invasive *Aspergillus* and *Candida* infections.

It has a wide range of antifungal activity against various fungal strains such as *Candida* spp. resistant to older azoles, *Cryptococcus neoformans*, *Aspergillus* spp., *Rhizopus* spp, *Bacillus dermatitidis*, *Candida immitis, Histoplasma capsulatum* and other opportunistic filamentous and dimorphic fungi [55–59]. Posaconazole is available only for oral administration and has a bioavailability of 8–47% on an empty stomach, which increases by 400% with the ingestion of a fatty meal [60]. The drug is primarily metabolized by the liver, approx. 77% of the unaltered drug is excreted in the faeces and small amount in the urine [61].

The efficacy of posaconazole is mostly in the treatment of zygomycosis, invasive fusariosis, cryptococcal meningitis, coccidioidomycosis and other central nervous system fungal infections [62]. The main limitation of posaconazole is that no i.v. formulation is available so it cannot be used in some severely ill patients. It has few common side effects such as nausea, vomiting, headache, abdominal pain and diarrhoea [63].

**Ravuconazole**

**Figure 12:**
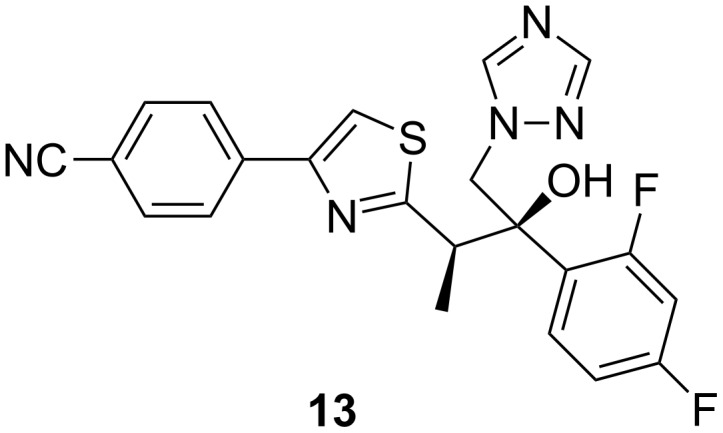
Ravuconazole (**13**).

Ravuconazole (**13**, Figure 12) is a triazole currently undergoing phase II clinical trials. It is highly active against a wide range of fungi, including *Candida* spp., *Candida neoformans* and other yeast species as well as isolates that are resistant to fluconazole. An in vitro study evaluating the activity of ravuconazole against 923 clinical isolates of non-dermatophyte filamentous fungi indicated that the drug is highly active against *Aspergillus* spp. and has inhibitory activity against other species of hyaline filamentous fungi and zygomycetes and black moulds. In this study, ravuconazole was active against 56.2% of mucorales tested, whilst itraconazole was active only against one-third of the isolates and voriconazole was inactive against almost the whole collection of mucorales [64].

Ravuconazole is highly (>95%) protein bound, shows linear pharmacokinetics over the anticipated dosage range and undergoes hepatic metabolism. It has a significant potential for drug–drug interactions through the cytochrome P450 enzyme system.

### Spectrum of antifungal activity and resistance of fungal pathogens

The triazoles are active against C*andida albicans*, non-albicans *Candida* spp., *Cryptococcus neoformans* and the dimorphic fungi. Among non-albicans *Candida* spp., they are less active against *Candida glabrata* and inactive against *Candida krusei* with the exception of voriconazole and some investigational triazoles. Various *Aspergillus* spp. and dematiaceous moulds are only affected by itraconazole and voriconazole. Voriconazole is also active against *Fusarium* spp. The main reason for resistance to antifungal triazoles is due to differences or alterations in the composition of membrane associated sterols, alterations in the biosynthetic pathway of ergosterol, genetic changes in the enzyme by mutation, over expression, gene amplification etc. Secondary resistance has been observed by prolonged exposure to azoles in chronic recurrent oropharyngeal candidiasis, allogeneic hematopoietic stem-cell transplantation, and chronic granulomatous disease. Other secondary resistance has been observed in HIV infected patients with chronic recurrent oropharyngeal candidiasis [65–67].

### Other applications of triazoles

Triazoles have been suggested as water replacements in proton conductors used in fuel cells. Once doped into membranes, these triazoles improve the conductivity of the membranes under anhydrous conditions. Due to their amphoteric nature, these compounds act as proton acceptors and donors. Their amphoteric nature and their mobility, especially at high temperature, are the two main reasons for these materials to be considered for water replacement. However, to prevent them from washing out of the membrane, small molecules must be immobilized. 4,5-Dicyano-1*H*-1,2,3-triazole (DCTz) showed proton conductivity in the order of 1 mS/cm in dry conditions at 100 °C for composites of 4,5-dicyano-1*H*-1,2,3-triazole with polyacrylonitrile in the absence of any other external proton sources [68].

Some triazole derivatives such as 4-amino-5-mercapto-3-methyl-1,2,4-triazole (AMMT), 4-amino-5-mercapto-3-ethyl-1,2,4-triazole (AMET) and 4-amino-5-mercapto-3-propyl-1,2,4-triazole (AMPT) have been evaluated as new corrosion inhibitors for the corrosion of muntz alloy (Cu:Zn 60:40) in acidic and neutral solutions [69]. The efficiency of inhibitors depends on the nature and the state of the metallic surfaces, chemical composition and structure of the inhibitor [70].

Novel heteroleptic iridium complexes containing the 1-substituted-4-phenyl-1*H*-1,2,3-triazole cyclometalating ligand were synthesized by the [3 + 2] Huisgen dipolar cycloaddition method which was utilized to prepare a class of bidentate ligands by adding different substituents to the triazole nucleus. By a judicious choice of ligands, a library of new luminescent ionic iridium complexes was prepared [71].

3-Amino-1,2,4-triazole is an inhibitor of mitochondrial and chloroplast function. Commercial grade 3-amino-1,2,4-triazole is used as a herbicide and cotton defoliant [72].

The triazole derivatives such as S-3307, S-3308, triadimefon, and paclobutrazol are recommended for use both as fungicides and plant growth regulators [73].

### Future prospects

The antifungal agents available on the market have various drawbacks such as toxicity, narrow spectrum of activity and fungistatic profiles rather fungicidal, and some also exhibit drug–drug interactions. In view of the high incidence of fungal infections in immune compromised patients, demands for new antifungal agents with a broad spectrum of activity and good pharmacokinetic properties have increased. The continuing demand for safe and effective broad spectrum antifungal agents with favorable pharmacokinetic properties has spurred both the design and development of new systemically active antifungal triazoles. Even with the advent of ketoconazole, the search for improved antifungal azole agents has continued due in part to concerns over the potential for toxicity and poor penetration into cerebrospinal fluid associated with ketoconazole. Fluconazole is the current drug of choice for treatment of severe infections caused by *Candida* species and *Candida neoformans.* Fluconazole has only weak activity against isolates of *Aspergillus* species [minimum inhibitory concentration (MIC) values of 400 μg/ml], since the drug has low potency (IC_50_ = 4.8 μM) against lanosterol 14α-demethylase. The development of the earlier triazole compounds which included SCH 39304 (genoconazole), SCH 42427 (saperaconazole) and Bay R 8783 (electrazole) had to be discontinued as a result of safety concerns. SCH 56592 is a potent antifungal triazole with excellent in vitro activity against *Aspergillus* spp*.* and *Candida* spp [42]*.* Another promising triazole, D0870, a derivative of fluconazole, exhibited significant variations in plasma pharmacokinetics besides having weak anti-aspergillus activity. Other fluconazole derivatives in different stages of development include voriconazole and ER 30346 (BMS 207147). BMS 207147 is very potent antifungal against *Candida* spp. *Candida neoformans, Aspergillus fumigates* and *Trichosporon beigelii.* Voriconazole also shows non-linear pharmacokinetics but has raised some concern with respect to its ocular toxicity. ER 30346 shows the best anti-aspergillus activity, SCH 56592 is a hydroxylated analogue of itraconazole with potent in vitro and in vivo activity [74–75].

## Conclusion

The azole antifungal drugs have introduced a new era in antifungal chemotherapy. Although they all act through a similar mechanism, they vary widely in fungal spectrum, pharmacokinetics and toxicities. Other potent agents in earlier stages of development may further expand the options available in this remarkable group of compounds. Recent advances in antifungal chemotherapy and the addition of newer broad spectrum triazoles, offer clinicians more effective and less toxic alternatives to conventional amphotericin B. Even with the introduction of azole antifungal drugs and despite recent advances, mortality rates from invasive fungal infections remain high and there is a necessity for new treatment options. Earlier diagnosis, rapid restoration of the host immune system, the combination of antifungals and development of other compounds may improve the outcome of IFIs.
